# Substrate Type and Free Ammonia Determine Bacterial Community Structure in Full-Scale Mesophilic Anaerobic Digesters Treating Cattle or Swine Manure

**DOI:** 10.3389/fmicb.2015.01337

**Published:** 2015-11-30

**Authors:** Jiabao Li, Junpeng Rui, Minjie Yao, Shiheng Zhang, Xuefeng Yan, Yuanpeng Wang, Zhiying Yan, Xiangzhen Li

**Affiliations:** ^1^Key Laboratory of Environmental and Applied Microbiology, Chengdu Institute of Biology, Chinese Academy of SciencesSichuan, China; ^2^Environmental Microbiology Key Laboratory of Sichuan Province, Chengdu Institute of Biology, Chinese Academy of SciencesSichuan, China; ^3^Department of Chemical and Biochemical Engineering, College of Chemistry and Chemical Engineering, Xiamen UniversityFujian, China

**Keywords:** anaerobic digester, animal manure, bacterial community, free ammonia, core community

## Abstract

The microbial-mediated anaerobic digestion (AD) process represents an efficient biological process for the treatment of organic waste along with biogas harvest. Currently, the key factors structuring bacterial communities and the potential core and unique bacterial populations in manure anaerobic digesters are not completely elucidated yet. In this study, we collected sludge samples from 20 full-scale anaerobic digesters treating cattle or swine manure, and investigated the variations of bacterial community compositions using high-throughput 16S rRNA amplicon sequencing. Clustering and correlation analysis suggested that substrate type and free ammonia (FA) play key roles in determining the bacterial community structure. The COD: ${\text{NH}}_{4}^{+}-\text{N}$ (C:N) ratio of substrate and FA were the most important available operational parameters correlating to the bacterial communities in cattle and swine manure digesters, respectively. The bacterial populations in all of the digesters were dominated by phylum Firmicutes, followed by Bacteroidetes, Proteobacteria and Chloroflexi. Increased FA content selected Firmicutes, suggesting that they probably play more important roles under high FA content. Syntrophic metabolism by Proteobacteria, Chloroflexi, Synergistetes and Planctomycetes are likely inhibited when FA content is high. Despite the different manure substrates, operational conditions and geographical locations of digesters, core bacterial communities were identified. The core communities were best characterized by phylum Firmicutes, wherein *Clostridium* predominated overwhelmingly. Substrate-unique and abundant communities may reflect the properties of manure substrate and operational conditions. These findings extend our current understanding of the bacterial assembly in full-scale manure anaerobic digesters.

## Introduction

Anaerobic digestion (AD) represents an efficient process for the treatment of various kinds of organic waste along with biogas production (Alvarado et al., 2014). The biological process involves four sequential steps: substrate hydrolysis, fermentation, acetogenesis and methanogenesis, which requires the cooperation of bacteria and archaea (Ali Shah et al., 2014). Archaea, especially methanogens, are key players during methanogenesis, thus attracting much attention. However, bacterial populations are essential in anaerobic digesters treating insoluble organic materials, such as animal manure, since the hydrolysis step is often the bottleneck of AD process due to the nature of complex and recalcitrant substrates (Werner et al., 2011; St-Pierre and Wright, 2014; Carballa et al., 2015). In addition, bacteria also take charge of the critical syntrophic metabolism coupled to methanogenesis (Morris et al., 2013), so that stable performance can be achieved during the AD processes.

Multiple factors including digester design, substrate and operational conditions influence microbial community structures (Lin et al., 2013; Town et al., 2014). Substrate is recognized as a key factor affecting fermentation efficiency, as well as the microbial community composition (Zhang et al., 2013; Ziganshin et al., 2013). Cluster analysis of the bacterial and archaeal communities shows that reactors treating similar substrates group together (Sundberg et al., 2013). It is postulated that substrate type determines the observed differences in phylogenetic structure based on a meta-analysis of 16S rRNA gene sequences retrieved from 79 digesters treating various substrates (Zhang et al., 2014). Nonetheless, microbial populations in anaerobic manure digesters can display high variations even at the digestion of a common core substrate (St-Pierre and Wright, 2014).

Operational conditions including temperature and ammonia content could impact bacterial community structure. It is reported that bacterial communities clustered based on factors rather than the input materials in lab-scale thermophilic digesters (Town et al., 2014). That is probably because high temperature imposes much stronger influences than other operational conditions on the communities (Ziganshin et al., 2013). Animal manure is widely used as substrate in anaerobic digesters, which often contains high free ammonia (FA) due to high protein content (Deublein and Steinhauser, 2008; Riviere et al., 2009). FA has an inhibiting or even toxic effect on prokaryotic communities because it may passively diffuse into cells, causing proton imbalance and potassium deficiency (Sprott and Patel, 1986; Chen et al., 2008). FA also inhibits pH sensitive species (Chen et al., 2008). Syntrophic acetate oxidization (SAO) performed by SAO bacteria is observed to become important under high ammonia content (Schnurer et al., 1999; Schnurer and Nordberg, 2008). Therefore, the selectivity of ammonia to different microbial populations could be the mechanism structuring prokaryotic communities in anaerobic digesters treating animal manure.

In anaerobic digesters, core communities [represented by operational taxonomic units (OTUs)] are commonly found in different digesters with relative high abundances (Riviere et al., 2009; Saunders et al., 2015). In addition, core communities of anaerobic digesters were found within microbial populations that are capable of performing substrate hydrolysis, fermentation and syntrophic metabolism (St-Pierre and Wright, 2014; Rui et al., 2015). They may vary depending on different substrate (Riviere et al., 2009; Nelson et al., 2011; St-Pierre and Wright, 2014). Therefore, the elucidation of core and unique communities among different full-scale anaerobic digesters might be useful to indicate important traits of AD process, and to identify putatively important organisms for microbial management in AD (Saunders et al., 2015). Previously, core and unique OTUs were identified in 7 different full-scale anaerobic digesters with the clone library method (Riviere et al., 2009). Three anaerobic digesters shared 132 core OTUs (St-Pierre and Wright, 2014). However, information is still limited due to limited samples of full-scale biogas reactors. Core and unique OTUs can be better determined by using more independently-operated full-scale anaerobic digesters and high-throughput methods (Saunders et al., 2015). In China, there are 3717 large-scale (digester volume >500 m^3^) and 18,853 medium-scale (digester volume of 50–500 m^3^) biogas plants that have been established by the end of 2009 (Jiang et al., 2011). Of these, swine and cattle manure are two most popular substrates. Few studies have been conducted to identify the potential core and unique bacterial populations, as well as the factors driving the assembly of the bacterial communities, among multiple full-scale anaerobic digesters treating animal manure.

In this study, we collected 20 sludge samples from independently-operated full-scale anaerobic digesters at different geographical locations across China. The objectives were to identify: (i) important factors shaping the bacterial communities, and (ii) the potential core and unique bacterial populations in digesters treating cattle and swine manure.

## Materials and methods

### Sample description and operational parameters

From August to October, 2012, 20 sludge samples (at least 400 ml each) were collected from digesters located from the northeast to the southwest of China (Table S1), including 8 cattle manure digesters (c1–c8) and 12 swine manure digesters (s9–s20). Autoclaved anaerobic flasks were filled with sludge samples that discharged from the sampling valve, and transported to the laboratory on ice immediately. Most digesters were built at livestock breeding plants for the treatment of animal manure. Operational parameters, e.g., digester type and volume, substrate type, sludge retention time (SRT), biogas production and digestion temperature, were directly obtained from the plant operators. Chemical properties of sludge, including pH, chemical oxygen demand (COD), ionized-ammonia (${\text{NH}}_{4}^{+}-\text{N}$), and phosphate, were measured according to the method described previously (Li et al., 2014; Shen et al., 2014). Free ammonia (FA) was calculated based on the total ammonia, pH and temperature values (Rajagopal et al., 2013).

Among the 20 sampling digesters, continuous stirred-tank reactors (CSTRs) were used by 18 digesters, and upflow solid reactors (USRs) were used by the rest 2 digesters (c6 and s9). Operational parameters varied, with SRT ranging between 15 and 30 days, digester volume between 60 and 10,000 m^3^, biogas production between 0.13 and 1.0 m^3^ m^−3^ d^−1^, digester temperature between 25 and 36.5 °C, sludge pH between 6.50 and 7.75, COD between 314.70 and 7243.80 mg l^−1^, ${\text{NH}}_{4}^{+}-\text{N}$ between 89.24 and 3474.14 mg l^−1^, FA between 1.34 and 149.23 mg l^−1^, and phosphate between 2.97 and 92.39 mg l^−1^.

### DNA extraction and 16S rRNA gene amplicon sequencing

Genomic DNA was extracted according to the method of Rademacher et al. (2012). The concentrations and quality of DNA were checked using a NanoDrop 2000 spectrophotometer. For 16S rRNA gene amplicon sequencing, the primers 515F (5′-GTGYCAGCMGCCGCGGTA- 3′) and 806R (5′- GGACTACHVGGGTWTCTAAT -3′) were used to amplify V4-V5 region of the 16S rRNA gene. The forward and reverse primers had modifications introduced at 5′ ends to contain the Miseq sequencing adaptor sequences. The 10-mer barcode sequence was added between the adaptor and reverse primer sequences. An aliquot of 10 ng of purified DNA from each sample was used as a template for PCR amplification in 25 μl reaction mixture. The following conditions were used: denaturation at 94°C for 3 min, followed by 30 cycles of denaturation at 94°C for 30 s, annealing at 55°C for 1 min and extension at 72°C for 1 min, with a final extension at 72°C for 5 min. Triplicate PCR reactions were performed per sample and pooled. The PCR products were purified using Gel Extraction kit (Omega bio-tek). Equal molar of PCR product from each sample was pooled together. Sequencing library was constructed using Truseq DNA Library Prep kits according to the manufacture's instruction and sequenced by Illumina Miseq platform using MiSeq Reagent Kit v2.

### Sequence data analysis

The raw sequences were sorted based on the unique sample barcodes, trimmed for sequence quality using the QIIME pipeline (Caporaso et al., 2010). Chimera sequences were removed using the Uchime algorithm (Edgar et al., 2011). Each sample was rarefied to an equal sequencing number of 11,080 (the fewest number of sequences in a single sample). The sequences were clustered by the complete-linkage clustering method incorporated in the QIIME pipeline (Caporaso et al., 2010). Operational taxonomic units (OTUs) were picked at 97% identity using cd-hit in the QIIME pipeline. Singleton sequences were filtered out. Shannon's diversity index, Chao1 estimator of richness and the observed OTUs number were calculated at 97% sequence identity in the Ribosomal Database Project (RDP) pipeline (http://pyro.cme.msu.edu/). A phylogenetic affiliation of each representative sequence was analyzed by RDP Classifier at a confidence threshold of 80% (Wang et al., 2007).

After reprocessing, potential core, substrate-unique and shared communities (represented by specific OTUs) were identified. Core OTUs were distributed in all the digesters, while substrate-unique OTUs only existed in more than 80% of cattle or swine manure digesters. Shared OTUs were distributed in more than 80% of all digesters and excluded the core OTUs. Based on the abundance-based differences, shared OTUs were further categorized into three types: cattle-abundant (higher relative abundance in cattle manure digesters), swine-abundant (higher relative abundance in swine manure digesters), and both-equal OTUs (equal abundance in both cattle and swine digesters).

### Statistical analysis

The overall differences in the bacterial community structures were evaluated by principal coordinates analysis (PCoA) based on Bray-Curtis distances using the relative abundances of OTUs without singletons as the input data. Three nonparametric multivariate permutation tests, including multiple response permutation procedure (MRPP), permutational multivariate analysis of variance (Adonis), and analysis of similarity (ANOSIM), were performed to assess the significance of the difference in the structures of bacterial communities between the two types of digesters (Deng et al., 2012). Using a set of OTUs without singletons, Canonical correspondence analysis (CCA) was performed to discern the correlations between the bacterial communities and the operational parameters. FA, pH, ${\text{NH}}_{4}^{+}-\text{N}$, COD, C:N were selected by the R function *bioenv* as the most significant parameters. The above analyses were carried out with the Vegan package in R (Dixon, 2003). Pearson's correlation analysis was conducted to examine the correlations between the community composition and the operational parameters with SPSS Statistics 21.0.

### Nucleotide sequence accession numbers

The original sequencing data are available at the European Nucleotide Archive by accession No. PRJEB6969 (http://www.ebi.ac.uk/ena/data/view/PRJEB6969).

## Results

### Diversity and structure of bacterial communities

The variations of bacterial community composition within 20 full-scale anaerobic digesters were characterized using barcoded amplicons resulting in 429,907 chimera-free reads and further 4629 OTUs at a cutoff of 97% similarity. The 744 OTUs had an average relative abundance of more than 0.01%. The bacterial diversity indices varied across all the samples (Table S2). Correlation analysis showed significant and negative relationships between Shannon's index, the observed number of OTUs and free ammonia (FA), sludge pH and ${\text{NH}}_{4}^{+}-\text{N}$ (Table 1). Other parameters including biogas production, C:N, digester volume, temperature, SRT, COD, and phosphate did not show any significant correlations with the diversity indices.

**Table 1:** **Pearson's correlation between operational parameters and bacterial diversity indices of all samples^a^**.

**Diversity indices**	**pH**	**${\text{NH}}_{4}^{+}-\text{N}$**	**FA**
Chao1 richness	−0.44	−0.37	−0.35
Observed OTUs	−0.62^**^	−0.67^**^	−0.52^*^
Shannon's index	−0.56^**^	−0.57^**^	−0.62^**^

a *FA, free ammonia*.

** *P < 0.01*,

* *P < 0.05*.

*Only operational parameters with significant relationships with any of the three indices were listed*.

**Figure 1:**
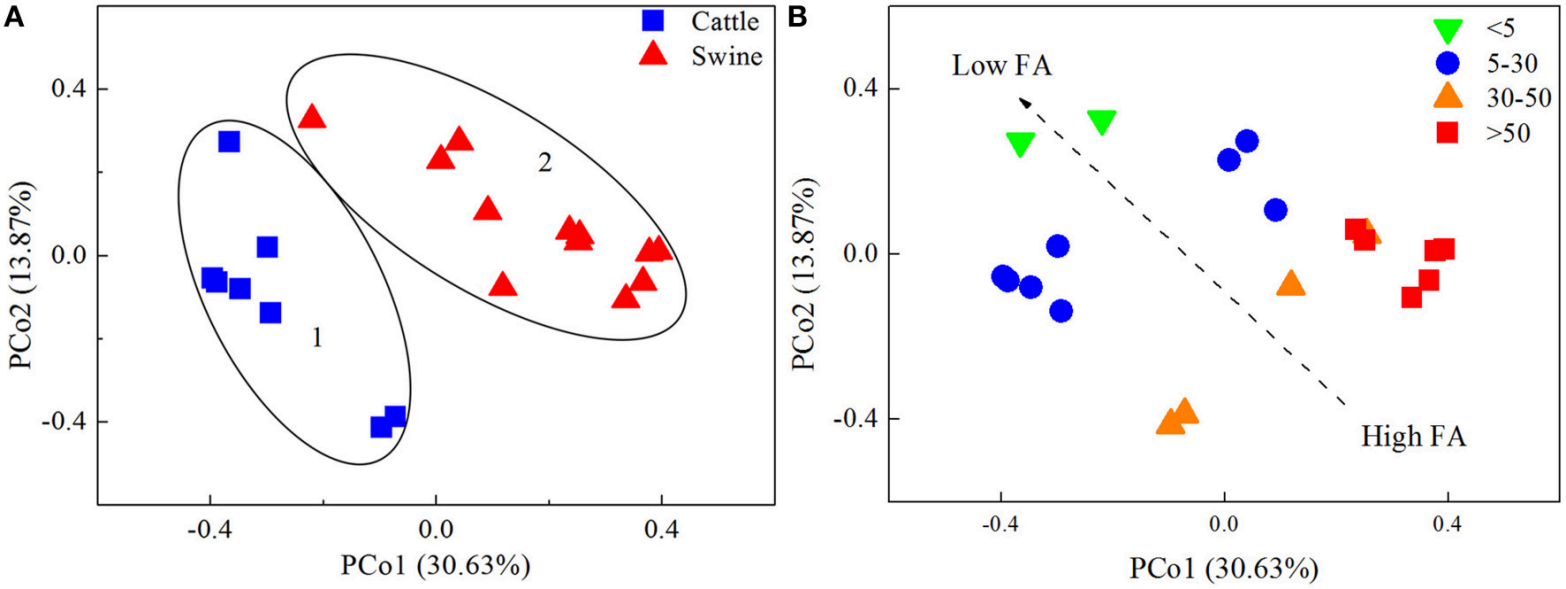
**The principal coordinates analysis (PCoA) plots of the bacterial communities in 20 independently-operated full-scale anaerobic digesters**. Bray-Curtis distance was used for the PCoA analysis. Plots were ranked by **(A)** substrate type, and **(B)** free ammonia content (FA, mgl^−1^).

Two potential clusters were observed by means of PCoA analysis of the bacterial communities (Figure 1A). Cluster 1 contained 8 samples exclusively from cattle manure digesters; Cluster 2 consisted of 12 samples originating from swine manure digesters. Approximately, PCo1 and PCo2 explained 45% of the total variations in the bacterial community structure among the digesters. The significant difference between the two potential clusters was verified with three nonparametric multivariate permutation tests (Adonis, *F* = 5.85, *P* = 0.001; ANOSIM, *R* = 0.77, *p* = 0.001; MRPP, δ = 0.64, *P* = 0.001). The results indicated that substrate type was likely to segregate bacterial communities in these anaerobic digesters. In addition, the segregation of bacterial communities within each cluster was also observed, suggesting that other parameters also contributed to the variations in the bacterial communities.

### Bacterial community composition

**Figure 2:**
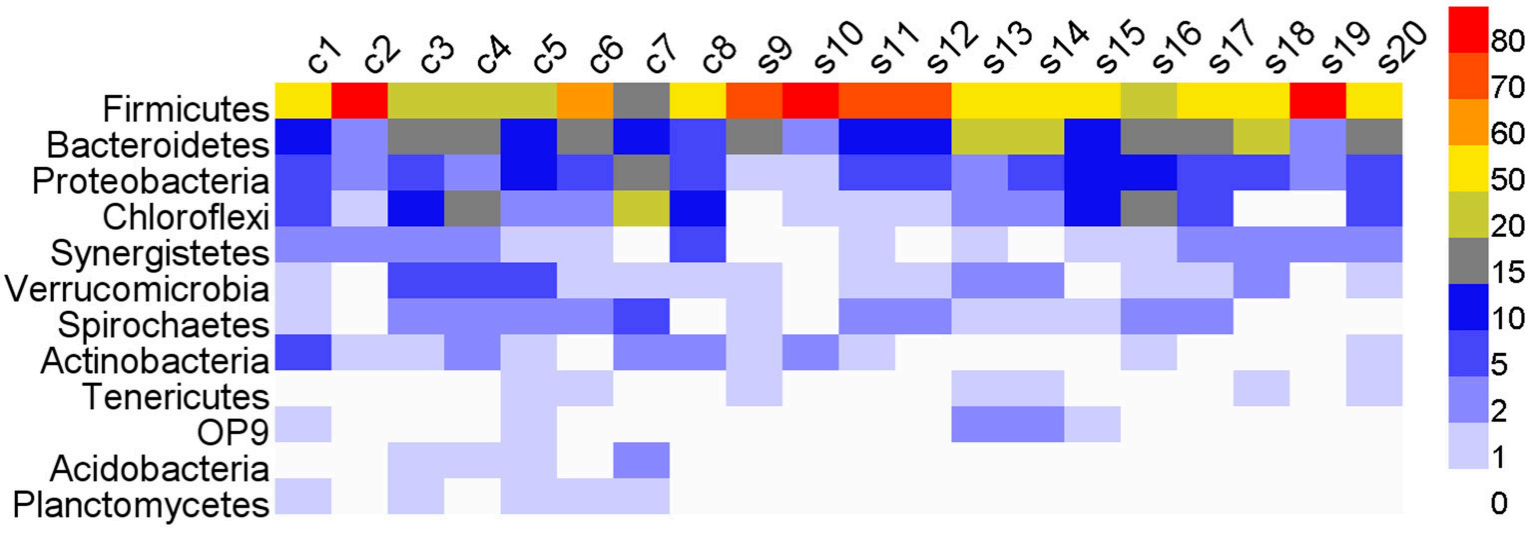
**The heatmap showing the relative abundances of various phyla (>0.1%) across all the digesters**. c, cattle manure digesters; s, swine manure digesters.

Roughly, 99% of total reads were annotated at phylum level, 93% at order level, and 45.5% at genus level. In all the 20 digesters, the phylum Firmicutes (57.79%) was dominated, followed by Bacteroidetes, Proteobacteria and Chloroflexi (Figure 2 and Table S3). Above taxa constituted 85.6% of total reads. Other phyla were relatively low in the relative abundance in most digesters (Figure 2 and Table S3). All the phyla shared high degree of variations in the relative abundances in different samples. Notably, Firmicutes overwhelmingly dominated in one cattle manure digester and five swine manure digesters (Table S3). However, Chloroflexi predominated in digester c7 (23.88%), followed by Spirochaetes and Acidobacteria.

**Figure 3:**
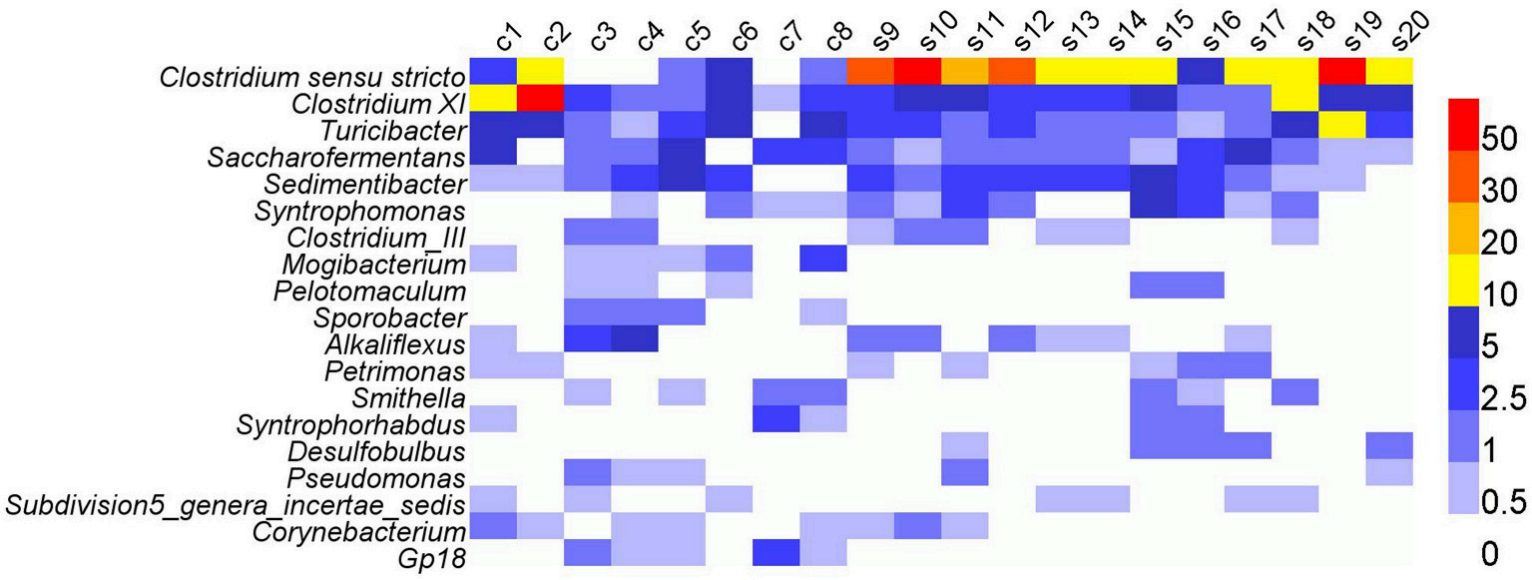
**The heatmap showing the relative abundances of abundant genera (>0.3%) across all the digesters**. c, cattle manure digesters; s, swine manure digesters.

At the genus level, most abundant genera (relative abundance >0.30%) were affiliated to phylum Firmicutes, e.g., *Clostridium sensu stricto, Clostridium* XI, *Syntrophomonas, Clostridium_*III, and *Pelotomaculum* (Figure 3 and Table S3). Other abundant genera were also included, such as *Smithella, Syntrophorhabdus* in phylum Proteobacteria, and *Corynebacterium* in phylum Actinobacteria. The 19 genera accounted for 40.4% of total genera reads. Despite the dominance of these genera, high variations in the relative abundance were observed in different samples, e.g., *Clostridium sensu stricto* varied between 0.14 and 56.75%, and *Clostridium* XI between 0.89 and 52.15% (Table S3).

### Potential core, substrate-unique, and shared communities

Based on the occurrence and the relative abundances of OTUs in all the samples, we defined three major groups.

#### Core OTUs

OTUs distributing in all the 20 digesters were defined as core OTUs. This study identified 25 core OTUs that made up 3.36% of 744 abundant bacterial OTUs, but accounted for 34.4% of total reads (Table S4). The core OTUs were primarily affiliated to genera *Clostridium sensu stricto, Clostridium* XI, *Turicibacter, Saccharofermentans, Sedimentibacter, Syntrophomonas* in phylum Firmicutes, order Bacteroidales of phyla Bacteroidetes, genus *Acinetobacter* in phylum Proteobacteria, family Anaerolineaceae in phylum Chloroflexi, genus *Subdivision5_genera_incertae_sedis* in phylum Verrucomicrobia and genus *Corynebacterium* in phylum Actinobacteria. The relative abundances of most core OTUs were higher than 0.5% (Table S4).

#### Substrate-unique OTUs

Substrate-unique OTUs were defined as those merely distributed in more than 80% of cattle or swine manure digesters. Nineteen and twenty OTUs were detected only in cattle and swine manure digesters, consisting of 5.62 and 3.29% of their respective total reads (Table S4). These OTUs were mainly distributed in phylum Firmicutes, and also in phylum Actinobacteria in Cluster 1, and phylum Bacteroidetes in Cluster 2. However, most of them were unclassified at the genus level.

#### Shared OTUs

Shared OTUs were defined as those found in more than 80% of each type of digesters, but excluding the core OTUs. The identified 108 shared OTUs made up 21.49% of total reads, and can be further binned into three groups: cattle-abundant, swine-abundant and both-equal OTUs. There were 14 and 9 OTUs as cattle- and swine-abundant OTUs, respectively (Table S4). Swine-abundant OTUs were all represented by phylum Firmicutes. Differently, cattle-abundant OTUs included phyla Firmicutes, Synergistetes, Bacteroidetes, Chloroflexi and Actinobacteria.

### Relationships between bacterial community compositions and available operational parameters

**Figure 4:**
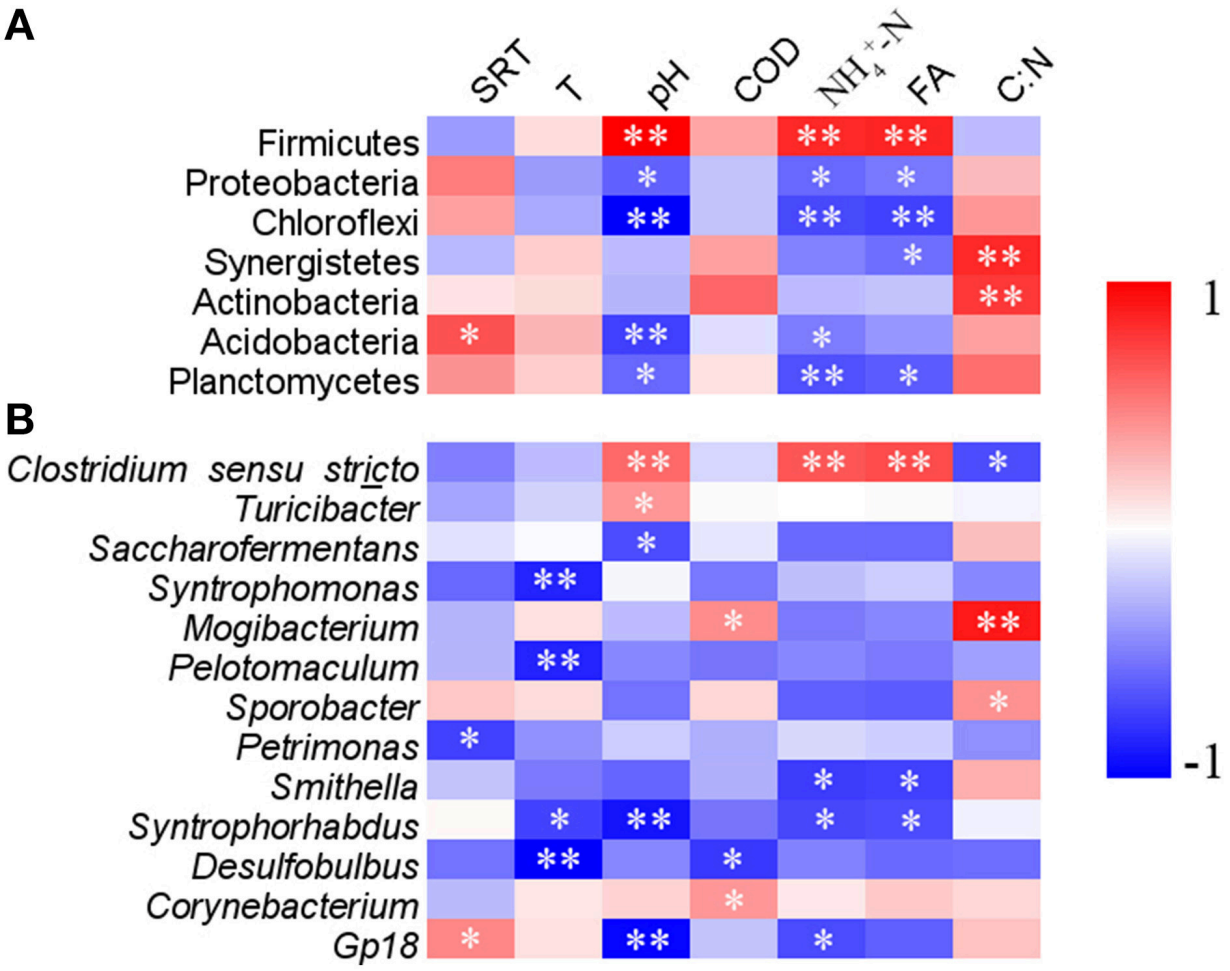
**The heatmap showing the correlations of relative abundance of various phyla (A) and genera (B) with available operational parameters**. The phyla and genera with average relative abundances of higher than 0.1% and 0.3%, respectively, were listed. SRT, sludge retention time; T, digester temperature; COD, chemical oxygen demand; FA, free ammonia; C:N, ratio of COD/${\text{NH}}_{4}^{+}-\text{N}$. **p < 0.01, *p < 0.05.

Correlation analysis of the bacterial community compositions with most available operational parameters showed that biogas production, temperature, COD and phosphate did not significantly correlate to the relative abundances of any dominant phyla (Table S5). However, FA positively correlated with the relative abundance of phylum Firmicutes, and negatively correlated to Proteobacteria, Chloroflexi, Synergistetes, and Planctomycetes (Figure 4A). Sludge pH and ${\text{NH}}_{4}^{+}-\text{N}$ usually showed same correlation patterns with these phyla as those of FA. Sludge pH and SRT were also significantly correlated with the relative abundance of Acidobacteria. C:N was positively correlated with the relative abundances of phyla Synergistetes and Actinobacteria (*p* < 0.01).

At the genus level, sludge pH, FA, and ${\text{NH}}_{4}^{+}-\text{N}$ were observed to correlate with 27, 24, and 24 genera, respectively (Table S6). Other operational parameters showed less correlation with the various genera. As the dominant genus, *Clostridium sensu stricto* was positively correlated with ${\text{NH}}_{4}^{+}-\text{N}$ and FA (*p* < 0.01), whereas *Smithella* and *Syntrophorhabdus* were negatively correlated with above two parameters (Figure 4B). Additionally, significantly positive correlations with sludge pH were observed for *Clostridium sensu stricto* and *Turicibacter*, but negative correlations between pH and *Saccharofermentans* and *Syntrophorhabdus*.

**Figure 5:**
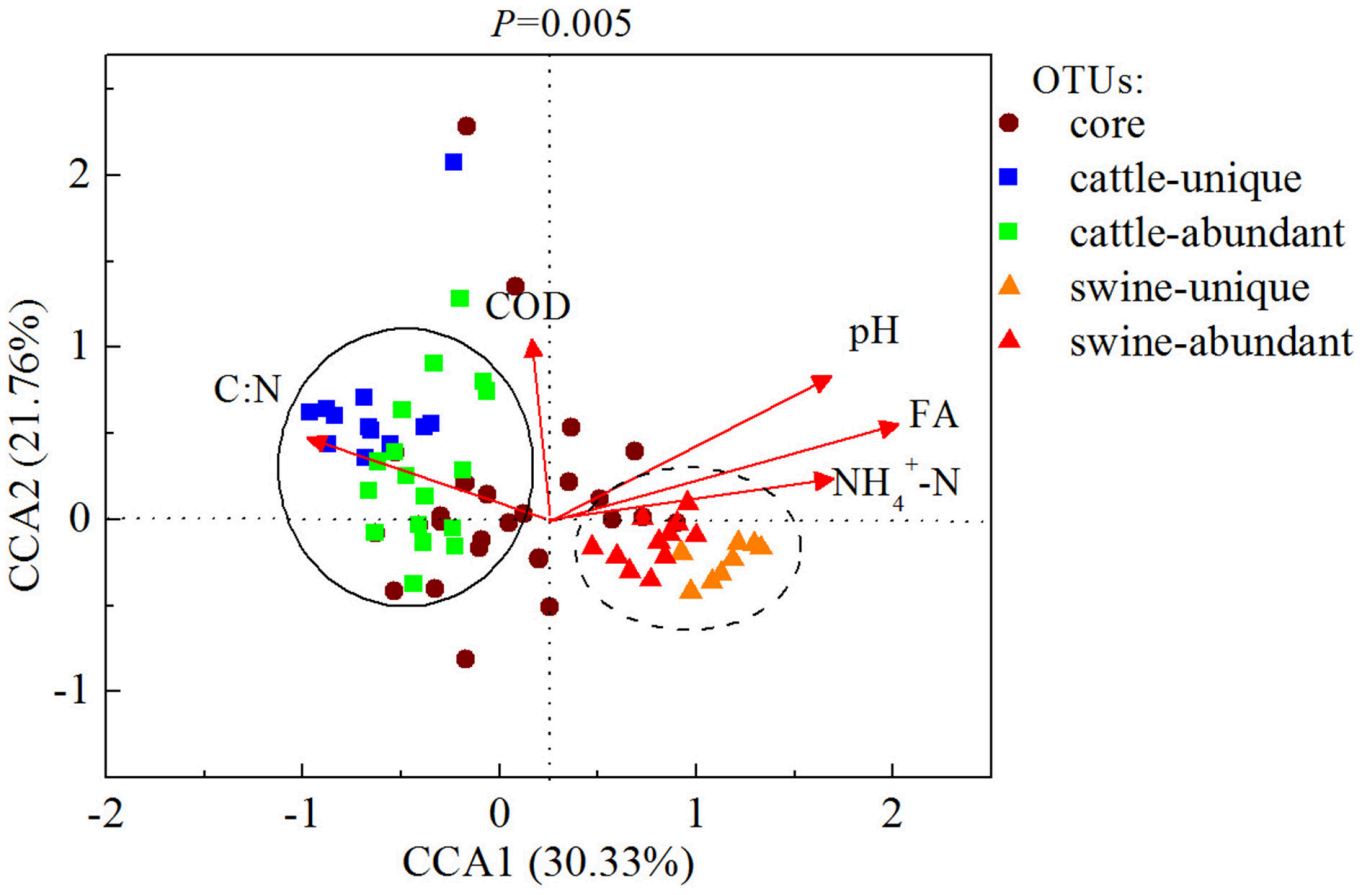
**Canonical correspondence analysis (CCA) of the bacterial communities and the operational parameters in 20 independently-operated full-scale anaerobic digesters**. OTUs with relative abundance of higher than 0.01% and 5 significant operational parameters selected by bioenv were applied for CCA. The core, substrate-unique and substrate-abundant OTUs were listed. COD, chemical oxygen demand; FA, free ammonia; C:N, ratio of COD/${\text{NH}}_{4}^{+}-\text{N}$.

At the OTU level, ${\text{NH}}_{4}^{+}-\text{N}$, FA, C:N, and pH were the four most dominant available parameters that were correlated with 39, 35, 30, and 25 OTUs, respectively, in all digesters (Table S7). Cattle-abundant and unique OTUs were exclusively and positively correlated with C:N and COD (*p* < 0.05; Table 2), while swine-abundant and unique OTUs were significantly correlated with FA and ${\text{NH}}_{4}^{+}-\text{N}$. These results were further corroborated with CCA analysis (Figure 5).

**Table 2:** **Pearson's correlation of substrate-unique and abundant OTUs with operational parameters^a^**.

	**OTU ID**	**pH**	**COD**	**${\text{NH}}_{4}^{+}-\text{N}$**	**FA**	**Phosphate**	**C:N**	**Taxa rank**
Cattle-unique	1334						0.46^*^	Lachnospiraceae
	1390		0.51^*^				0.91^**^	Clostridiales
	4218		0.55^*^				0.95^**^	Clostridiales
	4402		0.46^*^				0.82^**^	Lachnospiraceae
	5948		0.54^*^				0.82^**^	Lachnospiraceae
	8380		0.54^*^				0.79^**^	Lachnospiraceae
	9822						0.76^**^	Coriobacterineae
	10378		0.53^*^				0.92^**^	Clostridia
	11301						0.52^*^	Clostridiales
	12256		0.5^*^				0.89^**^	Clostridiales
	14799		0.49^*^				0.90^**^	Coriobacterineae
Cattle-abundant	532	−0.53^*^		−0.45^*^			0.55^*^	*Sporobacter*
	4023		0.53^*^					Corynebacterineae
	5640						0.52^*^	Bacteria
	5752		0.46^*^				0.63^**^	Planococcaceae
	6375			−0.46^*^			0.77^**^	Firmicutes
	7332		0.5^*^				0.55^*^	Corynebacterineae
	7857						0.65^**^	Bacteria
	8408		0.56^**^				0.72^**^	*Clostridium sensu stricto*
	8614	−0.57^**^						*Anaerovorax*
	11860	−0.45^*^		−0.49^*^	−0.49^*^		0.56^**^	Ruminococcaceae
Swine-unique	1558	0.47^*^		0.92^**^	0.90^**^			Firmicutes
	3161			0.63^**^	0.62^**^	0.55^*^		Clostridiales
	7786			0.65^**^	0.70^**^			*Lactobacillus*
	9568			0.55^*^	0.49^*^			Bacteria
	11072			0.78^**^	0.84^**^			Firmicutes
	13212	0.52^*^			0.54^*^			*Clostridium sensu stricto*
	13626			0.55^*^	0.49^*^			Clostridiaceae 1
	14200			0.73^**^	0.76^**^			Bacteroidetes
	15022			0.56^*^	0.56^**^			*Lactobacillus*
Swine-abundant	1099	0.48^*^		0.79^**^	0.80^**^			Syntrophomonadaceae
	1351			0.45^*^	0.56^**^			Lachnospiraceae
	2441					0.45^*^		Clostridiales
	4394	0.56^**^					−0.48^*^	*Clostridium* XI
	5383			0.54^*^				Clostridiales
	8677			0.63^**^	0.52^*^			Bacteria
	13608	0.46^*^		0.57^**^	0.61^**^			*Clostridium sensu stricto*

a *COD, chemical oxygen demand. FA, free ammonia. C:N, ratio of COD/${\text{NH}}_{4}^{+}-\text{N}$*.

** *P < 0.01*,

* *P < 0.05. Only significant relationships were listed*.

## Discussion

Although our studied digesters were operated under different operational conditions, substrate types and geographical locations, potential core and unique OTUs were identified. Further, substrate type and free ammonia (FA) were revealed as the most dominant factors differentiating bacterial communities in these digesters.

In this study, the observed clustering of the samples from different full-scale digesters could be attributed to substrate type. This is consistent with findings that substrate shapes microbial community structure in AD systems (Sundberg et al., 2013; Wagner et al., 2013; Ziganshin et al., 2013; Zhang et al., 2014). A wide variety of components in manure can be utilized to produce biogas in AD systems, e.g., protein, cellulose and lipid. Though the feedstock in both types of digesters is animal manure, different chemical natures and microbial communities in manure inoculums could contribute to the variations of community structure in the AD systems. Indeed, swine manure is a kind of protein-rich organic substrate (Hansen et al., 1998), while cattle manure is often composed of cellulose-rich material since the feedstock is mainly silage with high C:N (ASABE, 2005). In this study, cattle manure digesters did show significantly higher C:N compared to swine manure digesters (*p* < 0.05, data not shown).

Nevertheless, substrate type could not explain the observed segregation of the bacterial communities within both cluster members. A previous study also reported high variations of microbial populations in anaerobic manure digesters at the digestion of a common core substrate (St-Pierre and Wright, 2014). Operational parameters may cause such variations. In this study, correlation analysis revealed that pH, FA, and ${\text{NH}}_{4}^{+}-\text{N}$ were all significantly correlated with Shannon's diversity and the observed number of OTUs. However, further analysis revealed that sludge pH did not affect the clustering in both Cluster 1 and Cluster 2 samples. Rather, ${\text{NH}}_{4}^{+}-\text{N}$ and FA were highly related to the clustering of the samples (Figure 1B). FA is very toxic to methanogenic community (Chen et al., 2008). We also observed that the responses of different bacterial taxa to FA were not the same. Thus, the selection of different prokaryotic taxa by free ammonia is likely an important mechanism shaping prokaryotic community structure in manure AD systems.

Excessive FA is detrimental to AD process because high FA content not only changes pH in the digesters, but also causes proton imbalance and potassium deficiency in microbial cells (Sprott and Patel, 1986; Chen et al., 2008). In this study, though the sludge pH was neutral in these digesters, FA content highly varied, and more than 50 mg l^−1^ was detected in several swine manure digesters (s9, s10, s11, s12, s14, and s19). However, the FA content in the swine manure digesters is less likely to cause acute ammonia inhibition (Rajagopal et al., 2013). Alternatively, it may select specific bacterial populations that can better tolerate higher FA. For example, members of phylum Firmicutes, especially *Clostridium sensu stricto*, showed a positive correlation with FA (Figure 4 and Table S6). Firmicutes are ubiquitously involved in substrate hydrolysis, fermentation and acetogenesis (Nelson et al., 2011; De Vrieze et al., 2015). Several known species which are capable of syntrophic acetate oxidation (SAO) at elevated total ammonia concentrations are affiliated to this phylum (Schnurer et al., 1996; Westerholm et al., 2010; Sieber et al., 2012). Therefore, Firmicutes probably play more essential roles under high free ammonia content.

In contrast, many populations affiliated to Proteobacteria, Chloroflexi, Synergistetes, and Planctomycetes showed negative correlations with FA (*p* < 0.05; Figure 4A, Table S6), suggesting that they may be inhibited by high FA content. Many Synergistetes and Chloroflexi members are able to perform syntrophic metabolism in association with hydrogenotrophic methanogens during AD process (Sekiguchi et al., 2003; Yamada et al., 2006; Sieber et al., 2012). Dominant genera *Smithella* and *Syntrophorhabdus* in phylum Proteobacteria are able to convert propionate and aromatic compounds into acetate by syntrophic association with hydrogenotrophic methanogens (de Bok et al., 2001; Qiu et al., 2008). In this study, they were less represented in digesters with high FA content. In addition, other syntrophic microbes, e.g., *Pelotomaculum, Syntrophomonas*, and *Desulfobulbus* showed decreased trends, even though such changes were not significant at *p* = 0.05. The overall results indicated that syntrophic metabolism by these microbes are likely inhibited when FA content is high.

High FA content is also known to trigger the metabolic shift toward SAO (Schnurer et al., 1999; Schnurer and Nordberg, 2008). A limited number of mesophilic syntrophic acetate oxidizers have been isolated, e.g., *Clostridium ultunense* (Schnurer et al., 1996), *Syntrophaceticus schinkii* (Westerholm et al., 2010), and *Tepidanaerobacter acetatoxydans* (Westerholm et al., 2011). However, we did not observe the emergence of these species in most digesters, possibly suggesting that SAO is not a dominant pathway. This is likely caused by the fact that FA content (1.34–149.23 mg l^−1^) in our studied digesters did not reach the ammonia inhibition threshold (Hansen et al., 1998), and thus characterized SAO species were not observed. Alternatively, some uncharacterized heterogeneous SAO bacteria are possibly responsible for SAO in reactors with increased ammonia content (Werner et al., 2014). Further simultaneously in-depth studies of methanogenic and bacterial communities and their interactions are warranted.

The core communities play critical roles in AD processes and the concept might be useful to identify putatively important organisms for microbial management in AD (Saunders et al., 2015). The core bacterial communities were defined as those commonly found in anaerobic digesters, and six core OTUs were identified within phyla Chloroflexi, Betaproteobacteria, Bacteroidetes, and Synergistetes (Riviere et al., 2009). In line with St-Pierre and Wright (2014), we identified different core OTUs mainly distributed in phylum Firmicutes. This is probably due to different substrates used in anaerobic digesters, which support differential microbial populations in the engineered AD systems (Zhang et al., 2014). Indeed, phylum Chloroflexi may predominate in digesters treating municipal wastewater or sewage sludge (Riviere et al., 2009; Nelson et al., 2011; Sundberg et al., 2013), while Firmicutes are dominant in most manure digesters or co-digesters of mixed substrates (Liu et al., 2009; Sundberg et al., 2013; St-Pierre and Wright, 2014). As a result, *Clostridium* in phylum Firmicutes, which contains various genes encoding cellulose and hemicellulose-digesting enzymes for the degradation of complex plant fibers (Deublein and Steinhauser, 2008; Zhu et al., 2011), gained dominance in the core OTUs of these digesters. Other core OTUs mainly included genera *Turicibacter, Sedimentibacter, Saccharofermentans*, and *Syntrophomonas*. These bacterial populations were recognized as important players in substrate hydrolysis, fermentation, acetogenesis, and syntrophic metabolism (Bosshard et al., 2002; Chen et al., 2010; Vanwonterghem et al., 2014). Due to the combined high abundances of core communities in all the digesters, they may be targets for manipulation of microbial activities to achieve an efficient performance and stability in manure anaerobic digesters.

Substrate-unique and abundant OTUs were identified, while a majority of these OTUs were unclassified at genus level. Substrate-unique and abundant OTUs may reflect the variations of manure quality, and the differences in the digestive tracts of rumen and non-rumen animals. This is supported by the fact that cattle-unique and abundant OTUs were exclusively and positively correlated with C:N and COD (*p* < 0.05), while swine-unique and abundant OTUs positively correlated with FA and ${\text{NH}}_{4}^{+}-\text{N}$ (*p* < 0.05). The C:N and FA strongly select unique bacterial populations that can be well adapted in these anaerobic digesters.

Our findings are based on mesophilic digesters treating cattle and swine manure. When adding more digester samples with different substrates or operational parameters, such as chicken manure and high temperature, the revealed key factors shaping bacterial community structure may change. If one environmental factor outcompetes other factors, it may decouple the relationships between microbial communities and other factors (Rui et al., 2015). Indeed, temperature is recognized as a key factor to shift microbial community structure in AD systems (Sundberg et al., 2013; De Vrieze et al., 2015).

Overall, our study revealed that substrate and free ammonia play key roles in determining the bacterial community structure. The selection of different prokaryotic taxa by substrates and free ammonia is likely an important mechanism shaping prokaryotic community structure in manure AD systems. Core communities may be responsible for the central function in AD systems, while substrate-unique and abundant communities may reflect the selection effects largely exerted by substrate quality and operational conditions. These findings provide further understanding of the bacterial assembly in full-scale manure anaerobic digesters.

### Conflict of interest statement

The authors declare that the research was conducted in the absence of any commercial or financial relationships that could be construed as a potential conflict of interest.
